# Correction: Eliciting priors and relaxing the single causal variant assumption in colocalisation analyses

**DOI:** 10.1371/journal.pgen.1012155

**Published:** 2026-05-19

**Authors:** Chris Wallace

After publication of this article [1], concerns were raised regarding Figs 5 and S1. Specifically, Figs 5 and S1 appear similar to each other despite the Results of [1] stating that S1 Fig is an alternative example of where results are robust over a wide range of *p*_12_.

The corresponding author stated that S1 Fig is incorrect. The correct S1 Fig is provided with this notice.

There is also an error in the third sentence of the final paragraph of the Discussion section. The correct sentence is: The simulations here (Fig(4)) suggest that *p*_12_ = 5 × 10^−6^ provides a reasonable balance between power and false positive calls, but it is unlikely that any single point distribution on *p*_12_ captures all prior knowledge.

## Supporting information

S1 Fig Example of sensitivity analysis on a dataset which shows evidence for colocalisation at a predefined rule of posterior *P(H4)* > 0.5 across a wide range of *p*_12_. (PDF)
