# Supplementary material for: Eliciting priors and relaxing the single causal variant assumption in colocalisation analyses
Source: PLoS Genet. 2020 Apr 20;16(4):e1008720. doi: 10.1371/journal.pgen.1008720 (PMC7192519; doi:10.1371/journal.pgen.1008720)
Supplement: S1 Table — (PDF) [file pgen.1008720.s001.pdf]

Study	Data–type	p_1, p_2	p_{12}	Notes
[44]	eQTL–methQTL	empirical[1]	empirical[2]	
[27]	GWAS–eQTL	default	default	MS lead SNPs with eQTL signals; independent snps in each region
[43]	GWAS–molQTL	default	default	
[36]	GWAS–eQTL	default	default	
[25]	GWAS–methQTL	default	default	
[40]	GWAS–GWAS[3]	default	default	tested in 50kb windows around each of 6472 snps in HLA region
[24]	GWAS–chromQTL	default	empirical[4]	
[20]	GWAS–eQTL	default	default	post-TWAS;
[28]	GWAS–eQTL[5]	default	default	45 reQTL samples only
[29]	GWAS–eQTL	default	default	
[32]	GWAS–methQTL	default	default	
[26]	GWAS–eQTL			
[33]	GWAS–eQTL			
[35]	eQTL–pQTL			
[41]	GWAS–eQTL	gwas-pw[50]	gwas-pw[50]	implemented in coloc2 `https://github.com/Stahl-Lab-MSSM/coloc2`
[23]	GWAS–eQTL			
[31]	GWAS–eQTL	not stated	not stated	
[39]	GWAS–eQTL	not stated	not stated	used to confirm primary SMR analysis
[38]	GWAS–eQTL	default	default	
[21]	GWAS–eQTL	default	default	
[22]	GWAS–chromQTL	not stated	not stated	

Continued on next page

Table S1: Applied studies which used coloc in 2018

[1] set by #indep signals/#tests

[2] considered range of p12 and compared prior mean of H4 to posterior mean of H4

[3] pediatric vs adult disease

[4] The default prior on sharing ($P = 1 \times 10^5$) was used for all primary analyses, with priors $P = 1 \times 10^{-4}$ and $P = 1 \times 10^3$ evaluated separately with all other parameters unchanged.

[5] response eQTL

Study	Data–type	p_1, p_2	p_{12}	Notes
[37]	GWAS–pQTL	default	default	
[42]	eQTL–pQTL	estimated[6]	p12/(p12+p1)=0.75	used data to inform p1, p2. used conditioning and prior beliefs to inform p12
[30]	GWAS–eQTL	not stated	not stated	
[34]	GWAS–eQTL	default	default	

Table S1: Applied studies which used coloc in 2018. eQTL = expression QTL; mQTL = methylation QTL; pQTL = protein QTL; chromQTL = chromatin mark QTL; molQTL = selection of molecular QTLs

[6] #eqtl or #pqtl snps/number tested
