# Supplementary material for: Eliciting priors and relaxing the single causal variant assumption in colocalisation analyses
Source: PLoS Genet. 2020 Apr 20;16(4):e1008720. doi: 10.1371/journal.pgen.1008720 (PMC7192519; doi:10.1371/journal.pgen.1008720)
Supplement: S1 Text — (PDF) [file pgen.1008720.s002.pdf]

S1 Appendix

Outline of coloc method

Single SNP GWAS summary statistics, for a single trait, provide an estimate of the effect of that SNP on the trait, $\hat{\beta}$, and an estimate of the variance of $\hat{\beta}$, $\hat{V}$. Standard theory asserts that $\hat{\beta}$ is an unbiased estimate of the true effect of the SNP on the trait, β, such that

$$\hat{\beta} \sim N(\beta, \hat{V}).$$

We can calculate a Bayes factor to compare alternative hypothesis that β is non-zero to the null that $\beta = 0$.[1] It is convenient to specify a conjugate prior for β under the alternative, $\beta \sim N(0, W)$, where W is chosen by prior experience. For example, for a case-control study, it is common to set $W = 0.2^2$ which corresponds to a prior that $P(e^{|\beta|} > 1.2) < 0.05$ — i.e. that the odds ratio will exceed 1.5 with probability 0.05, but in the scale of data commonly used for GWAS, variations in the choice of W tend to have negligible effects on the Bayes factor. This leads to the single SNP, single trait Bayes factor

$$BF_1 = \frac{\int \pi(\hat{\beta}|\hat{\beta} \sim \mathcal{N}(\beta, \hat{V}))\pi(\beta|\beta \sim N(0, W))d\beta}{\pi(\hat{\beta}|\hat{\beta} \sim \mathcal{N}(0, \hat{V}))} = \frac{\pi(\hat{\beta}|\hat{\beta} \sim \mathcal{N}(0, \hat{V} + W))}{\pi(\hat{\beta}|\hat{\beta} \sim \mathcal{N}(0, \hat{V}))}. \quad (1)$$

Note that if we do not have $\hat{\beta}$, but we do have a p value, then we may estimate the variance of β, $\hat{V}$ as a function of sample size and MAF[3], and hence estimate $\hat{\beta} = Z\sqrt{\hat{V}}$ where Z is the Z score associated with p. In this case, the sign of $\hat{\beta}$ is not known, but that the sign of $\hat{\beta}$ is not required for what follows.

Assume now that we have two traits, 1 and 2, and are given the summary statistics at a single SNP X, $\hat{\beta}_1$, $\hat{\beta}_2$, $\hat{V}_1$, $\hat{V}_2$. For independent data sets, $corr(\hat{\beta}_1, \hat{\beta}_2) = 0$, making calculations simple. We can express the joint BF as in (1), if we write

$$\hat{\beta} = (\hat{\beta}_1, \hat{\beta}_2) \quad \text{and} \quad \hat{V} = \begin{pmatrix} \hat{V}_1 & 0 \\ 0 & \hat{V}_2 \end{pmatrix}. \quad (2)$$

Prior variance W of β can take different forms depending on the hypothesis under test at SNP X

$$
\begin{aligned}
H_0^X &: \beta_1 = \beta_2 = 0 \\
H_1^X &: \beta_1 \sim N(0, W_1),\ \beta_2 = 0 \\
H_2^X &: \beta_1 = 0,\ \beta_2 \sim N(0, W_2) \\
H_3^{X,Z} &: \beta_1 \sim N(0, W_1), \gamma_2 \sim N(0, W_2) \\
H_4^X &: \beta_1 \sim N(0, W_1), \beta_2 \sim N(0, W_2)
\end{aligned}
$$

where W_i is a prior on the effect size of a SNP on trait i if it is causal, and β_i, γ_i are used to denote the estimands for SNPs X and Z on traits i respectively.

Under a single causal assumption, it has been shown that

$$Pr(G|X \text{ causal}) = Pr(G_{-X}|G_X, X \text{ causal})Pr(G_X) = Pr(G_{-X}|G_X)Pr(G_X)$$

where G is the full genotype data, X is a SNP in G, and G_X, G_{-X} are used to represent the genotype data at SNP X and at all SNPs except X. [2] This assumption means the Bayes factor for X being causal and hypothesis H_0 being true can be written as

$$\frac{Pr(G_{-X}|G_X)Pr(G_X|\beta_1 \sim N(0, W_1))}{Pr(G_{-X}|G_X)Pr(G_X|\beta_1 = 0)} = \frac{Pr(G_X|\beta_1 \sim N(0, W_1))}{Pr(G_X|\beta_1 = 0)} \tag{3}$$

i.e. as a quantity which only depends on estimates at X. We can thus enumerate all possible causal SNP configurations of at most one causal variants per trait, and calculate the Bayes factor for each hypothesis as the sum of Bayes factors over all configurations consistent with it.[3]

These Bayes factors are then used to derive the posterior probabilities:

$$P(H_i|\text{Data}) \propto P(\text{Data}|H_i)P(H_i) \propto \frac{P(\text{Data}|H_i)P(H_i)}{P(\text{Data}|H_0)} = \text{BF}_i\, P(H_i)$$

where $P(H_i)$ and BF_i are used to denote the prior probability and Bayes factor for hypothesis H_i

respectively.

$|r_g|$ can be used to conservatively estimate $p_{12}\sqrt{q_1 q_2}$

We assume two traits Y_1, Y_2 can be modelled as

$$
\begin{aligned}
Y_1 &= \sum_{i=1}^{n_{12}} \alpha_i G_i + \sum_{i=1}^{n_1} \beta_i H_i + E_1 \\
Y_2 &= \sum_{i=1}^{n_{12}} \alpha_i' G_i + \sum_{i=1}^{n_2} \gamma_i J_i + E_2
\end{aligned}
$$

where i indexes variants, G_i are the n_{12} variants that contribute to both traits, H_i, J_i are n_1 and n_2 variants unique to traits Y_1, Y_2 and E_1, E_2 are residual non-genetic factors. We assume genotypes at all variants are independently sampled from the same distribution (iid)

$$G_i, H_i, J_i \overset{\text{iid}}{\sim} f$$

and that the effects of each variant H_i, J_i on the traits are also iid

$$\beta_i, \gamma_i \overset{\text{iid}}{\sim} N(0, W).$$

To allow for dependence of effects at variants G_i, we assume α_i, α_i' are sampled from a bivariate normal distribution

$$\begin{bmatrix} \alpha_i \\ \alpha_i' \end{bmatrix} \overset{\text{iid}}{\sim} MNV\left(\begin{bmatrix} 0 \\ 0 \end{bmatrix}, \begin{bmatrix} W & \rho W \\ \rho W & W \end{bmatrix} \right)$$

An extreme case might be that $\text{cor}(\alpha_i, \alpha_i') = \rho = 1$ so that $\alpha_i' = \alpha_i$. Then the genetic correlation between Y_1 and Y_2 is

$$r_g = \frac{\sum_i^{n_{12}} \alpha_i^2 \operatorname{var}(G_i)}{\sqrt{(\sum_i^{n_{12}} \alpha_i^2 \operatorname{var}(G_i) + \sum_i^{n_1} \beta_i^2 \operatorname{var}(H_i))(\sum_i^{n_{12}} \alpha_i^2 \operatorname{var}(G_i) + \sum_i^{n_2} \gamma_i^2 \operatorname{var}(J_i))}} \tag{4}$$

As all the variants and their effect estimates are iid from the same distributions, then

$$E(\alpha_i^2 \operatorname{var}(G_i)) = E(\beta_i^2 \operatorname{var}(H_i)) = E(\gamma_i^2 \operatorname{var}(J_i)) = \nu$$

and replacing each term in (4) by its expectation, we find, to a first order approximation,

$$E(r_g) \approx \frac{n_{12}\nu}{\sqrt{(n_{12}+n_1)\nu(n_{12}+n_2)\nu}} = \frac{n_{12}}{\sqrt{(n_{12}+n_1)(n_{12}+n_2)}} \tag{5}$$

The simplifying assumption that the shared variants have the same effect on the traits is obviously unrealistic, but different effects over the same number of shared variants would produce a smaller correlation, thus this is expected to be a conservative estimate. To see this, relax the assumption that $\rho = 1$, but assume instead that the fraction of genetic variance of each trait attributable to the total set of variants G_i, $i = 1 \dots n$ is fixed, then only the numerator of (5) will change, and is

$$E\left(\sum_i^{n_{12}} \alpha_i \alpha_i' \operatorname{var}(G_i)\right) = \sum_i^{n_{12}} E(\alpha_i \alpha_i')\nu/W) = \sum_i^{n_{12}} \rho w^2 \nu/W = n_{12}\rho\nu$$

Thus, $|r_g|$ is maximal if the effects of all variants G_i on the two traits are equal.

Using estimated functional proportion of the genome to set a lower bound of p_{12}

Let F be the event that a SNP is "functional" (in the sense that all causal variants for the traits considered are assumed to be members of the functional set of SNPs), and recall that A_1, A_2 are the events that a SNP is causal for traits 1 and 2 respectively. Because $A_i,\ i = 1..2$ occurs only when F occurs, $P(A_i \cap F) = P(A_i)$. We seek a lower bound for $p_{12} = P(A_1 \cap A_2)$, so assume $A_1 \perp\!\!\!\perp A_2 | F$, and denote $f = P(F)$. Then

$$
\begin{aligned}
p_{12} = P(A_1 \cap A_2) &= P(A_1 \cap A_2 | F) P(F) \\
&= P(A_1|F) P(A_2|F) P(F) \\
&= \frac{P(A_1 \cap F)}{P(F)} \frac{P(A_2 \cap F)}{P(F)} P(F) \\
&= \frac{P(A_1) P(A_2)}{P(F)} \\
&= \frac{q_1 q_2}{f}
\end{aligned}
$$

References

[1] Wakefield J. Bayes factors for genome-wide association studies: comparison with P -values. Genetic Epidemiology. 2009;33(1):79–86. doi:10.1002/gepi.20359.

[2] Giambartolomei C, Vukcevic D, Schadt EE, Franke L, Hingorani AD, Wallace C, et al. Bayesian test for colocalisation between pairs of genetic association studies using summary statistics. PLoS Genet. 2014;10(5):e1004383.

[3] Maller JB, McVean G, Byrnes J, Vukcevic D, Palin K, Su Z, et al. Bayesian refinement of association signals for 14 loci in 3 common diseases. Nature genetics. 2012;44(12):1294.
