# Supplementary figures and images for: Eliciting priors and relaxing the single causal variant assumption in colocalisation analyses
Source: PLoS Genet. 2020 Apr 20;16(4):e1008720. doi: 10.1371/journal.pgen.1008720 (PMC7192519; doi:10.1371/journal.pgen.1008720)

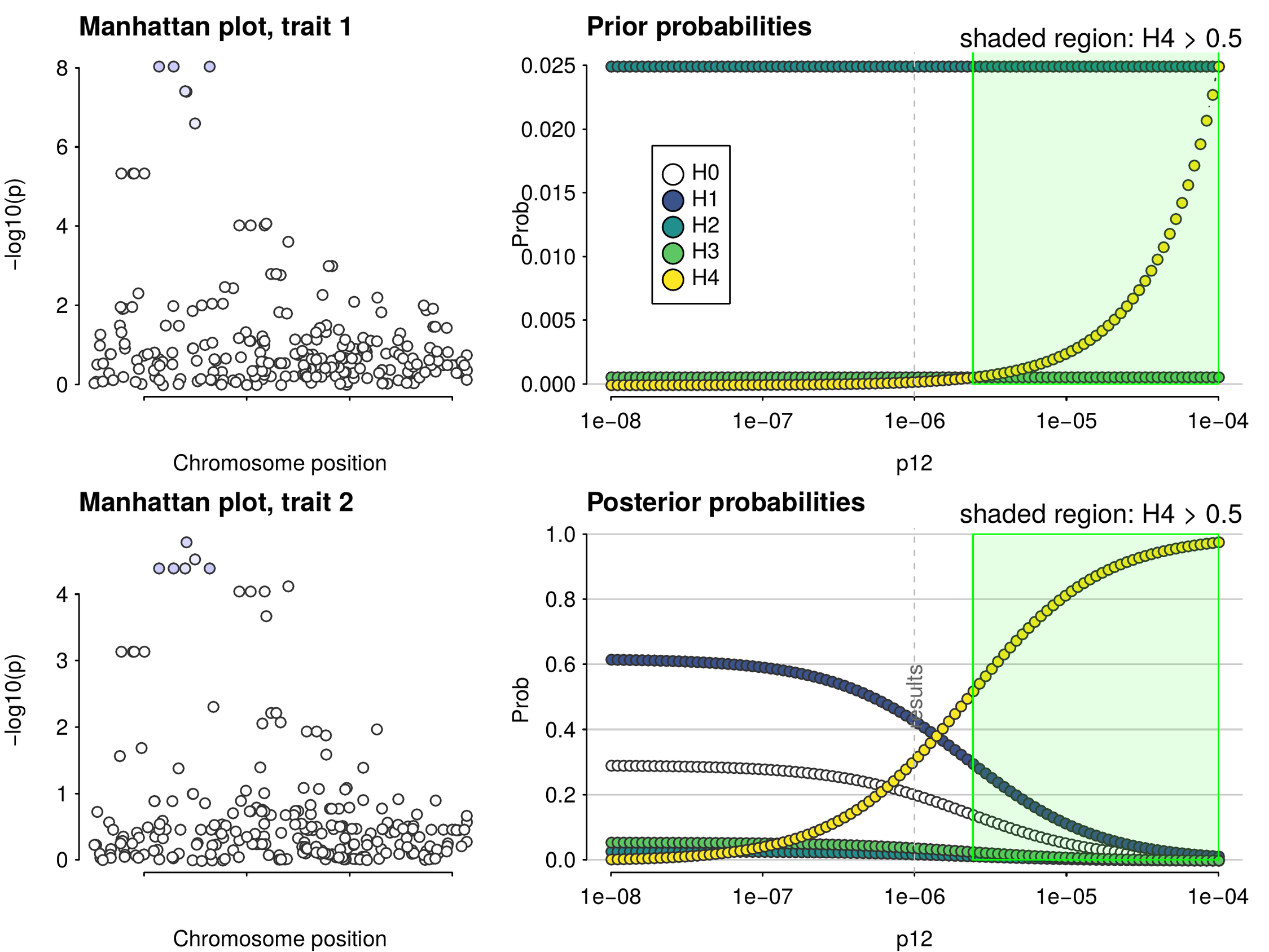

Supplement: S1 Fig — (TIF) [file pgen.1008720.s003.tif]

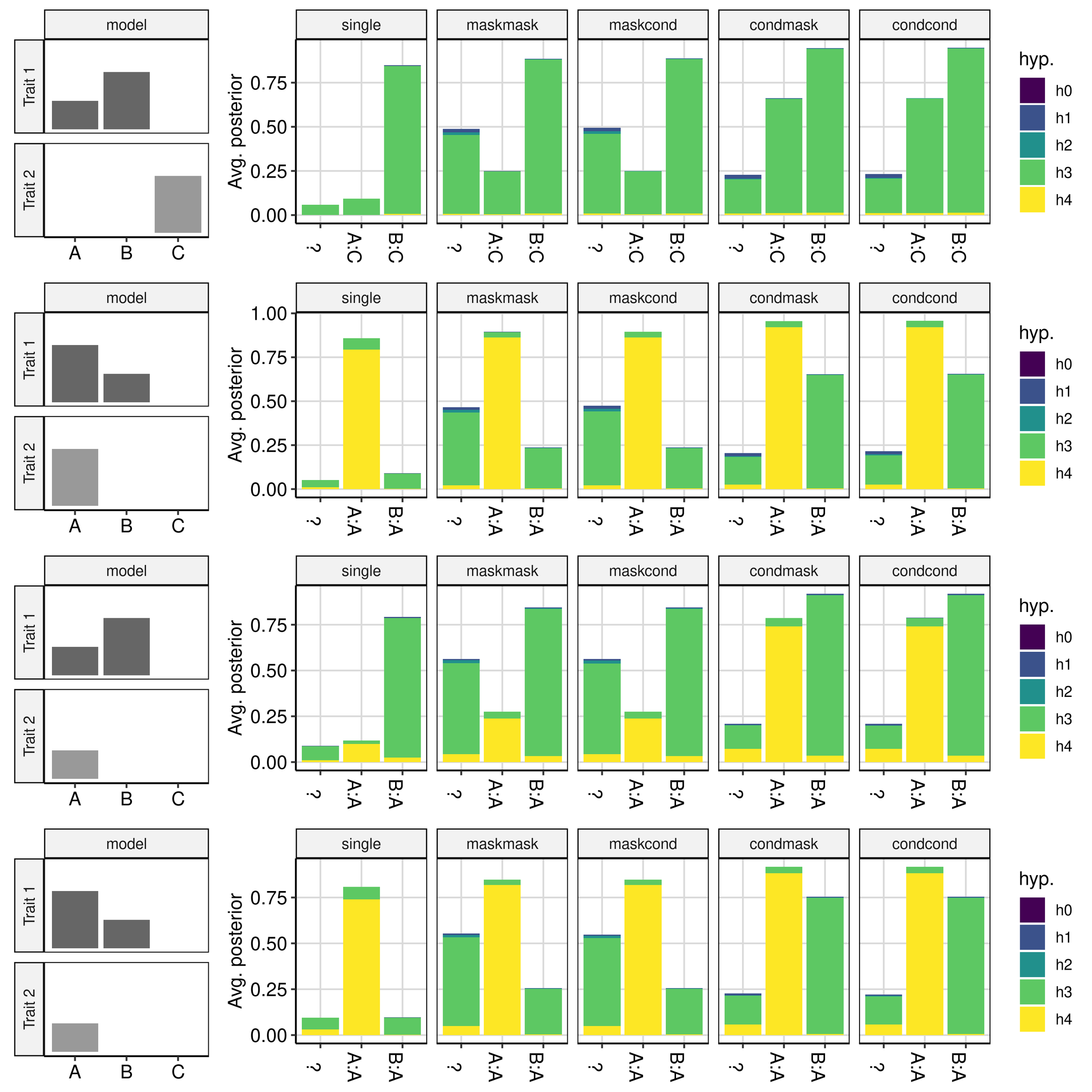

Supplement: S2 Fig — (TIF) [file pgen.1008720.s004.tif]

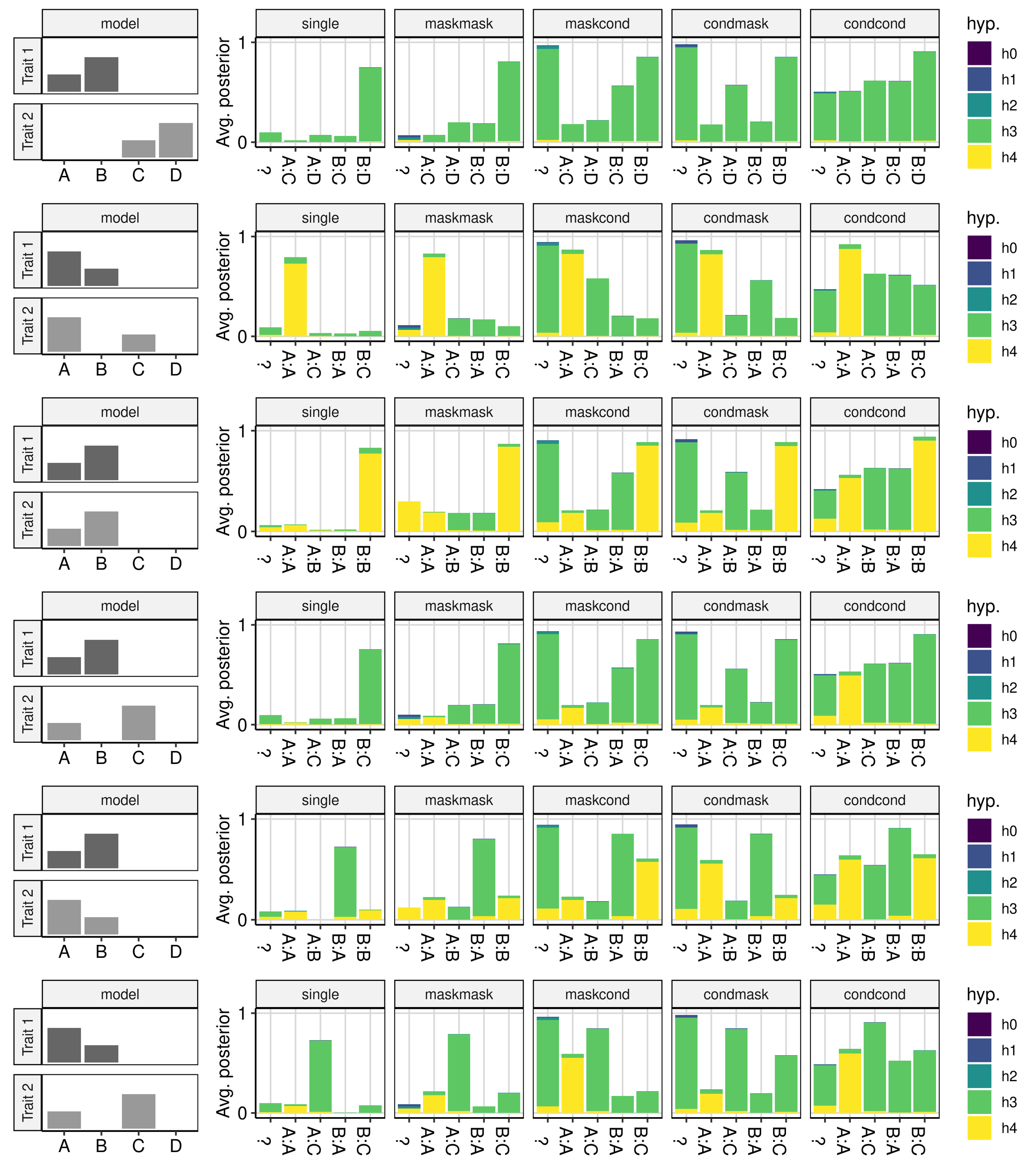

Supplement: S3 Fig — (TIF) [file pgen.1008720.s005.tif]
