# Supplementary material for: Eliciting priors and relaxing the single causal variant assumption in colocalisation analyses
Source: PLoS Genet. 2020 Apr 20;16(4):e1008720. doi: 10.1371/journal.pgen.1008720 (PMC7192519; doi:10.1371/journal.pgen.1008720)
Supplement: Attachment — Submitted filename: coloc response.pdf [file pgen.1008720.s006.pdf]

I thank reviewers for helpful and thoughtful comments. Each point is addressed individually below, with the exception of two points raised by different reviewers.

First, the suggestion that I provide some form of **default choice of priors** for users was common to all three.

My explicit intention writing this was to stress the importance of considering priors on a case-by-case basis rather than adopting any defaults. However, as reviewer 2 says, this may have the inadvertent consequence of prior-hacking. Therefore I have added the following new text in the Discussion:

> In summary, we find that coloc default values for the prior probabilities of single trait association, $p_1 = p_2 = 10^{-4}$, are well supported by data across a range of data types, but that the choice of p_{12} needs careful thought, and is expected to vary according to the pair of traits being considered. We recommend taking some time to do this before any analysis, documenting and justifying choices, using the coloc explorer app to translate between per-SNP and per-hypothesis values. The simulations here (Figure 4) suggest that $p_{12} = 5 \times 10^{-5}$ provides a reasonable balance between power and false positive calls, but it is unlikely that any single point distribution on p_{12} captures all prior knowledge. As varying p_{12} can sometimes have a substantial impact on inference, we strongly advise users to perform sensitivity analysis for key results. Both the justification of choices and the results of sensitivity analyses should be presented to accompany any published results.

I hope that this strikes a balance between requesting users take time to consider their choices, a suggestion of a not-unreasonable default, and a stronger suggestion that choices should be stated and justified to support any published inferences.

Second, both reviewers 1 and 2 asked for **more detail on masking**, and to put the concept in context by comparison with clumping and division of the genome into LD-independent blocks. I added the following to the Discussion:

> Here, we propose successively masking most associated SNPs and SNPs in LD with them. This has conceptual similarities to clumping, used in polygenic risk score construction to select the strongest signal in each LD-independent set of SNPs (Wray et al, 2014), and to the division of the genome into LD-independent blocks (Berisa et al, 2016), but differs to each. Our motivation is inverted compared to that for clumping: We aim to identify the set of SNPs whose GWAS summary statistics are likely to be unrelated to the masked signal, rather than select a single SNP from the masked group. We also select smaller sets of SNPs than found by dividing the genome into blocks, because we select SNPs only according to LD with the *sentinel*

SNP, rather than finding breakpoints such that *every SNP* in a block is likely to have minimal LD with any SNP outside that block.

Reviewer #1: *Colocalization is an increasingly important aspect of genetic fine mapping efforts (>60 papers in 2018) but, unusually in statistical genetics, the most popular software ("coloc") implements a Bayesian analysis with subjective priors. This paper demonstrates the potential sensitivity of coloc to the prior probability of colocalization, examines a huge amount of data to elicit suggestions for setting reasonable values, and provides software for performing sensitivity analysis. In addition, the assumption of one causal variant per region per trait is examined and a new approach, called masking, is suggested for situations in which current methods cannot be applied. Overall this paper gives useful guidance to users of coloc and provides insights into the method that should be of value.*

Minor comments

1. P4 L52 "ubiquity of genetic effects … concordant with an omnigenic model" – suggests that such ubiquity has been established when it remains a conjecture. Some rewording needed.

Reworded to

> However, the ubiquity of genetic effects on some measurable aspect of human physiology or health, which have prompted suggestions of an omnigenic model…

2. The Introduction starts off by introducing MR and appears to motivate colocalization primarily as a way to validate instruments in MR studies. But, as seen elsewhere in the paper, most of the applications are in delineating molecular pathways to disease. I'd suggest reworking the opening paragraph to better reflect the broader motivations for colocalization.

I have reworked paragraph 1 and the beginning of paragraph 2, as suggested:

> Assuming certain assumptions hold true, 5 this provides evidence that the first trait is somehow causal for the second. While MR was originally envisaged as a test of causality of specific risk factors for which tests of causality might be confounded in observational studies, MR has been extended to routinely assess the potential for any GWAS trait to mediate another.[6] However, the ubiquity of genetic effects on some measurable aspect of human physiology or health, which have prompted suggestions of an omnigenic model,[7] raise concerns that LD between causal variants can violate the MR assumption that the instrumental variable is only associated with the outcome through the "mediating" trait.[8] This routine testing of all possible mediators is similar in design to the assessment of potential molecular causes of disease, which has been addressed through alternative approaches that focus not on

> whether one trait is causal for another, but whether two traits share the same causal variants in a single, LD-defined, genetic region, termed colocalisation.
>
> While one such method is built on MR[9] and proceeds by filtering MR-positive associations via test of heterogeneity in the estimated proportional effect across multiple SNPs in the region, another popular colocalisation method, coloc,[10] avoids MR assumptions altogether. Instead, coloc enumerates every possible configuration of causal variants for each of two traits, and calculates ...

3. P7 L101 full text could be accessed for only 25 of 60 papers. Was this due to limitations of institutional subscriptions? Could not the corresponding authors provide manuscripts for research purposes?

In fact, the majority of the papers were excluded because they were methodological papers which reference coloc. I have revised the sentence for to make this clearer:

> We used Scopus to identify 60 papers which cited coloc and were published in 2018. Out of these, we extracted the subset of 25 papers that were both applied papers (rather than methodological) and for which full text could be accessed (Supp Table 1).

4. P7 L104 it would be interesting to know also how many papers used eCaviar or some other method to deal with multiple causal variants. Also, how often did the original discovery studies perform conditional analyses and rule out additional causal variants? So that when going to colocalization, the single causal variant assumption can be justified to some extent.

No studies used more than one colocalisation method. We have added mention of how many studies discussed the possibility of multiple or causal variants or using conditioning to adapt for them:

> Only four studies considered the potential for multiple causal variants in a region, either discussing the implications on their results, or using conditioning in at least one trait,

5. P7 L107 "prior probability … will depend" – should say "may depend" since at this point we haven't established this, and anyway since priors are subjective the user is free to believe that there is no dependence on the traits (but may then draw the wrong conclusion).

Corrected as suggested

6. P8 L124 “more likely” should be “relatively more likely”, otherwise this sentence is confusing. Initially I found this sentence counter-intuitive – seems that by looking at fewer SNPs we are more likely to find colocalization – but the point is that the prior probability of colocalization is higher relative to distinct variants when fewer SNPs are considered. However the lower number of SNPs would provide less evidence for colocalization so this is a false economy. Anyway some interpretation should be added to this and the previous paragraph as it is unclear what one should conclude from the observations.

Corrected to “relatively more likely” and added the following interpretation:

> This effect can be understood by noting that both H_3 and H_4 imply that each trait has exactly one causal variant in the region. Simple combinatorics implies that as the number of SNPs in a region increases, then the number of ways two different SNPs can be causal for the two traits (H_3) increases more rapidly than the number of ways one SNP can be causal for both (H_4). Hence, H_3 becomes relatively more likely than H_4 as the number of SNPs in the region increases.

7. P8 L132 note that all the estimates of p’s and q’s are based on statistically significant SNPs, and the number of truly associated variants must be larger. So the elicited priors must be lower bounds. What implications does this have for the final inferences?

I have added the following text after estimates of p and q values are discussed:

> Using conservative priors for p_1, p_2 in colocalisation analysis is likely to reduce power to detect either shared or distinct causal variants, because weaker signals may be wrongly interpreted as trait-unique or null. However, estimates from the largest available studies also represent at upper bound on the proportion of variants likely to be *detectably* associated in any new study from the same class of traits, and therefore relaxing the priors further might result in over-stating the evidence for causal variants and erring towards false detection of shared or distinct causal variants.

8. P9 L145 not clear how to get a posterior probability of association from just a prior and a p-value.

This is now explained in the first section of Methods, referring to quantities defined in the statistical description of coloc in the Appendix.

9. P10 L163, 165 the Appendix was not available to review.

Apologies, now attached.

10. P12 L208 "unlinked" -> "not in linkage disequilibrium". There is a difference between linkage and LD.

Corrected to "in linkage equilibrium".

11. P12 The masking method still needs an LD matrix, so the only real advantage over CoJo is that there is no need to align the alleles.

This is the advantage we state. I have now added the following to the discussion, which emphasises its potential use as a CoJo alternative that is more robust to small deviations in the estimated LD matrix:

> Masking is also likely to avoid substantial errors in the results of approximate conditioning that can occasionally result from small deviations from LD estimated in a reference population to that in the study sample, particularly when the reference population is smaller than that used to the GWAS (Benner et al, 2017).

12. P12 The masking method looks a lot like "clumping" as often used, for example, in constructing polygenic risk scores. Please clarify the difference, or use the same term to prevent jargon creep.

Please see response above.

13. P213 Figure 6 caption, "setting to 1 the Bayes factor" –the main text suggests setting the log Bayes factor to -3. Log in what base?

-3 was a typo, thank you for spotting it. I could correct it to "log Bayes factor to 0", but it is less confusing if I use the same scale as the main text, so I have corrected to "setting the per SNP Bayes factor to 1".

14. P14 L244 is it feasible to make the sensitivity analysis a default action in coloc, with the results being returned in the same object as the posteriors?

It would be, but I am reluctant to do this as often people run very many colocalisation analyses, and really we only care to check sensitivity for those that are "interesting" in some

way. Making it a default would impose a computational burden when not always needed. I have had long discussions with colleagues on this point before reaching this conclusion - it is not an obvious decision.

However, I would indeed like sensitivity analysis to become standard for any “interesting” results. Therefore I have added emphasis to the summary take-home message (see first response above).

15. P16 the Discussion would benefit from a summary take-home message, such as that the default values of p1 and p2 are OK but p12 needs more thought (and a summary of how to do this would also help).

See first response above.

Typos etc

1. P3 L32 “underly” -> “underlie”

corrected

2. P4 L59 “For example…” – the sentence has no active verb.

Reworded:

> First joint association of a SNP to gene expression and a GWAS trait is tested, then a test of heterogeneity in the estimated proportional effect across multiple SNPs in the region is used to assess whether the causal variant(s) for the two traits colocalise or are merely in LD.

3. P7 L117 delete “a”; change final “,” to “.”

corrected

4. P8 L140 the double “-“ is confusing, suggest just saying “to” or writing as an interval.

corrected

5. P9 L150 “One” -> “On”

corrected

6. P11 L178 in the equation below, can delete the intersection with A2 in the third expression.

corrected

7. P11 L180 spelling of “asymmetric”

corrected

8. P12 Figure 5 caption line 2, “belief” -> “beliefs”. What does the dotted line marked “results” mean?

Corrected, and added the following to the legend:

The dashed vertical line indicates the value of p_{12} used in initial analysis (the value about which sensitivity is to be checked).

9. P12 L212 “is” -> “are”

corrected

10. P14 L234 “are” -> “is”

corrected

11. P16 L288 “interpretable” -> “interpretation”

corrected

12. References are a bit sloppy, eg page numbers for refs 11 and 14.

corrected

13. Supplement P1, footnote 4 “P=1105” etc looks incorrect.

corrected

Reviewer #2: *This paper considers two important extensions to the currently most popular and influential colocalization method/software "coloc": a more suitable prior specification (than the current default) and relaxing the assumption of only one causal SNP. In particular, the first problem has been largely ignored in practice while its implication is significant, as the author has clearly shown in the paper. Although the proposed methods are not technically sophisticated, they can be tremendously useful as implemented in the "coloc" software. The paper was well written. I only have two very minor comments.*

Minor comments:

1. Prior elicitation is a well known and general problem in Bayesian statistics, both important and challenging. I agree with the author on all her points, and commend the author for providing a useful online tool "coloc explorer". However, without a "default" prior, I am not sure how useful it would be to a "typical" biologist without deep understanding of Bayesian statistics or "coloc" method; in fact, I would be a bit worried that someone might do "prior mining" to try to get more significant results. Some comments or guidelines might be helpful to a typical user.

Please see response above.

2. I completely agree with the author on both the advantages and limitations of the conditioning approach as compared to the proposed "masking" approach. However, if I understand correctly, with a typical small genomic region of interest, one would potentially mask out ALL SNPs in the region that are in LD with the lead SNP; in other words, is the new assumption simply that there is at most only one causal SNP in EACH LD block? If true, it is still like doing coloc analysis under the single causal SNP assumption for each LD block, which can be too restrictive given that there are only about two thousand (approximately independent) LD blocks in the huiman genome. Some clarifications and comments would be helpful.

Please see response above.

Reviewer #3: *This manuscript investigated how to derive data driven priors for best power of COLOC, provided a sensitivity analysis framework to assess the robustness of priors, and proposed a new masking approach for dealing with scenarios with multiple signals per*

region. It is very useful to provide guidelines for users of COLOC about how to setup priors to achieve the best power. However, this paper does not provide a clear guideline to readers. I have the following comments:

1) It would help refresh reader's mind if a brief description about the statistical procedure of the COLOC tool could be provided either in the Introduction section along with the five stated hypotheses, or at the beginning of the Results section.

I found I couldn't write a brief description that was clear, so I added a longer description as an additional section in the Appendix (pages 1-2), referenced from the Introduction.

2) I think it would be helpful to make a clear guideline table for readers, e.g., suggestive p1, p2, p12 prior values for a few different combinations of number of SNPs in the test region, total number of trait signals, if multiple signals exist in the test region. Or a such table could be provided for GTEx expression traits of different tissue types, which will provide readers a concrete example.

Please see response above.

3) It would be helpful if the authors could provide some descriptions about "coloc explorer" and "condmask coloc" and how to implement these two tools in the supplementary text.

Both tools are described at their location, and I am minded to leave it that way, because it will enable me to have a single source of documentation that I can update or clarify as needed. Instead I now link to both those sources (http://chr1swallace.github.io/coloc/articles/a04_sensitivity.html, http://chr1swallace.github.io/coloc/articles/a05_conditioning.html) in the manuscript where the tools are introduced, and hope this addresses your underlying concern.
