# Supplementary figures and images for: Correction: Eliciting priors and relaxing the single causal variant assumption in colocalisation analyses
Source: PLoS Genet. 2026 May 19;22(5):e1012155. doi: 10.1371/journal.pgen.1012155 (PMC13186361; doi:10.1371/journal.pgen.1012155)

**Manhattan plot, trait 1**

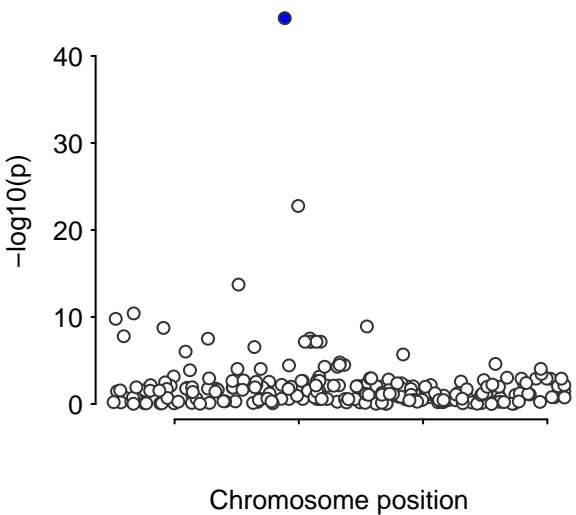

**Prior probabilities**

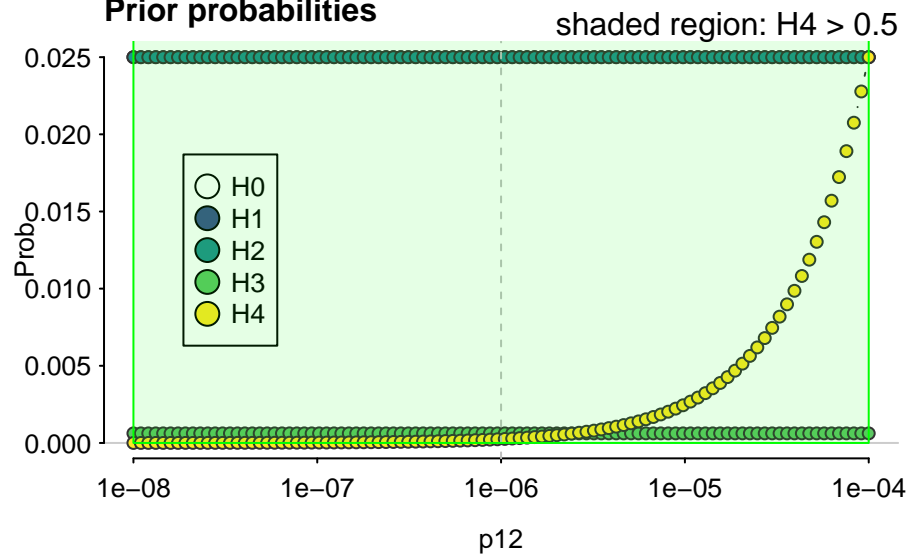

**Manhattan plot, trait 2**

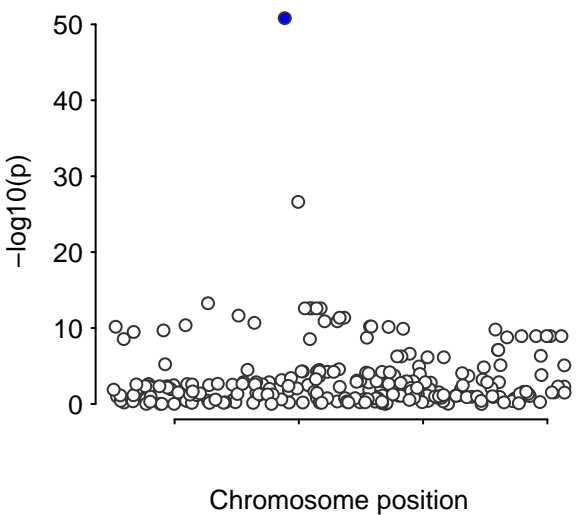

**Posterior probabilities**

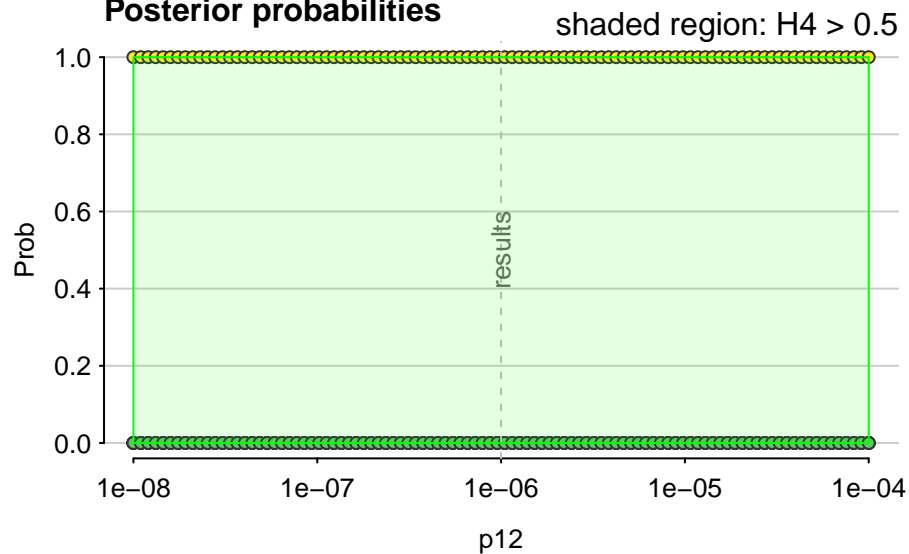

Supplement: S1 Fig — (PDF) [file pgen.1012155.s001.pdf]
